# Diabetes, Oxidative Stress, and DNA Damage Modulate Cranial Neural Crest Cell Development and the Phenotype Variability of Craniofacial Disorders

**DOI:** 10.3389/fcell.2021.644410

**Published:** 2021-05-20

**Authors:** Sharien Fitriasari, Paul A. Trainor

**Affiliations:** ^1^Stowers Institute for Medical Research, Kansas City, MO, United States; ^2^Department of Anatomy and Cell Biology, University of Kansas Medical Center, Kansas City, KS, United States

**Keywords:** diabetes, ROS, DNA damage, neural crest cell, craniofacial development

## Abstract

Craniofacial malformations are among the most common birth defects in humans and they often have significant detrimental functional, aesthetic, and social consequences. To date, more than 700 distinct craniofacial disorders have been described. However, the genetic, environmental, and developmental origins of most of these conditions remain to be determined. This gap in our knowledge is hampered in part by the tremendous phenotypic diversity evident in craniofacial syndromes but is also due to our limited understanding of the signals and mechanisms governing normal craniofacial development and variation. The principles of Mendelian inheritance have uncovered the etiology of relatively few complex craniofacial traits and consequently, the variability of craniofacial syndromes and phenotypes both within families and between families is often attributed to variable gene expression and incomplete penetrance. However, it is becoming increasingly apparent that phenotypic variation is often the result of combinatorial genetic and non-genetic factors. Major non-genetic factors include environmental effectors such as pregestational maternal diabetes, which is well-known to increase the risk of craniofacial birth defects. The hyperglycemia characteristic of diabetes causes oxidative stress which in turn can result in genotoxic stress, DNA damage, metabolic alterations, and subsequently perturbed embryogenesis. In this review we explore the importance of gene-environment associations involving diabetes, oxidative stress, and DNA damage during cranial neural crest cell development, which may underpin the phenotypic variability observed in specific craniofacial syndromes.

## Introduction

The vertebrate head and face comprise a complex assemblage of specialized tissues including the viscerocranium, chondrocranium and neurocranium, the central and peripheral nervous systems, and all of the major sense organs (Trainor, 2013). The anatomical complexity of the craniofacial complex coupled with the initiation of its development during early embryogenesis renders the head and face prone to malformation. In fact, of the 1% of all live births that present with a minor or major anomaly, about one-third affect the head and face (Gorlin et al., 1990). To date, more than 700 distinct craniofacial disorders have been identified and phenotypically described (Carey, 1992), and orofacial clefts (1:1,000) and craniosynostosis (1:2,500) represent two of the most common craniofacial birth defects. These disorders are characterized by a wide spectrum of anomalies with varying degrees of severity, and no phenotypes or syndromes are identical in all affected individuals. In fact, many affected individuals with extremely mild phenotypes go undiagnosed or are only diagnosed retrospectively upon the birth of a severely affected sibling or progeny (Trainor et al., 2009). Additionally, craniofacial anomalies can occur sporadically without a familial history of mutation, indicating that genetic background, environmental factors, and stochastic events can influence the etiology and pathogenesis of craniofacial disorders (Jones et al., 1975; Trainor et al., 2009; Bartzela et al., 2017). Therefore, a thorough understanding of the events controlling normal craniofacial morphogenesis is central to improving diagnosis and care for patients.

Craniofacial malformations typically arise due to defects in cranial neural crest cell formation, migration, or differentiation and are collectively termed “neurocristopathies.” Distinct and diverse phenotypes manifest depending on which phase of cNCC development is disrupted (Trainor, 2010; Watt and Trainor, 2014). Although variable gene expression and incomplete penetrance contribute to phenotypic variability, the impact of combinatorial genetic and non-genetic factors in craniofacial malformations is increasingly being recognized. A growing body of evidence demonstrates that neural crest cells are particularly sensitive to environmental influences such as diabetes and oxidative stress. Maternal diabetes is associated with an increased risk of birth defects (Kucera, 1971; Casson et al., 1997; Hawthorne et al., 1997; Von Kries et al., 1997; Mills, 2010) and may account for half of all perinatal deaths (Greene, 2001). In fact, women with pre-gestational diabetes have children with birth defects three to five times more frequently than women without diabetes (Greene, 2001). Oxidative stress-inducing teratogens, such as alcohol (Sulik et al., 1988), retinoic acid (Williams and Bohnsack, 2019), and nicotine (Zhao and Reece, 2005; Schneider et al., 2010), can also increase the likelihood of embryos born with craniofacial anomalies. Persistent oxidative stress can impinge on neural crest cell development through distinct mechanisms such as DNA damage, p53 activation and autophagy (Wang et al., 2015; Sakai et al., 2016; Han et al., 2019; Cao et al., 2020). Consistent with this idea, DNA damage and genome instability are associated with an increased incidence of cleft lip and/or palate (Kobayashi et al., 2013). Furthermore, mutations in DNA damage repair genes can result in craniofacial malformations, highlighting the importance of maintaining genome stability during normal craniofacial morphogenesis (Wong et al., 2003; Seeman et al., 2004; Altmann and Gennery, 2016; Sakai et al., 2016; Kitami et al., 2018; Boone et al., 2019; Yamaguchi et al., 2021). This led us to postulate that exogenous stressors, particularly oxidative stress and DNA damage, can worsen the damage caused by a particular neural crest cell disruptive mutation, thus exacerbating its phenotypic outcome. In this review, we provide a brief overview of cranial neural crest cell development and the effects of diabetes and oxidative stress on craniofacial morphogenesis. We will also discuss potential mechanisms for oxidative stress-induced DNA damage in modulating the phenotypic variability associated with craniofacial disorders.

## Neural Crest Cell and Craniofacial Development

**FIGURE 1:**
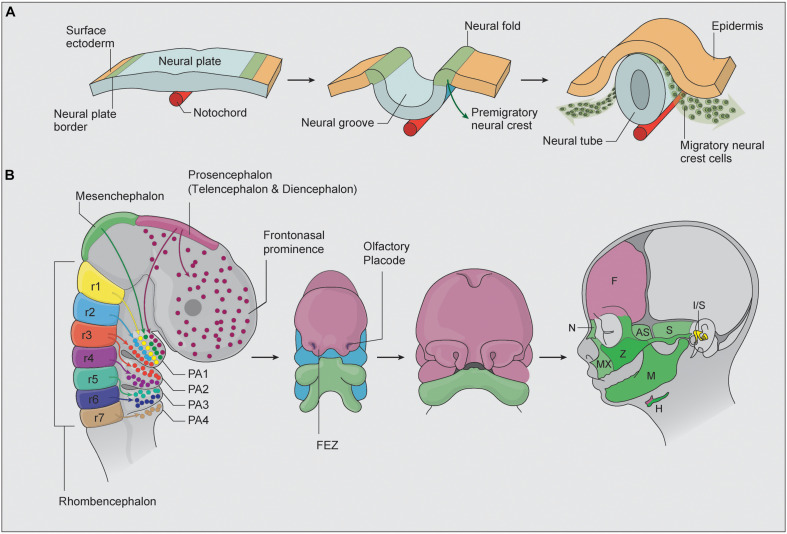
**(A)** NCC are initially specified within the neural plate border. As the two halves of the neural plate elevate to form a neural tube, NCC are induced and undergo EMT, after which they migrate and colonize the frontonasal prominences, first, second, third, and fourth pharyngeal arches (adapted from Simões-Costa and Bronner, 2015). **(B)** Cranial NCC patterns of migration and differentiation into the bone and cartilage of the head and face. During embryogenesis, the brain is specified into prosencephalon (diencephalon and telencephalon), mesencephalon, and rhombencephalon regions. The colors highlight regions of the developing face that correspond to NCC populations of different axial origins. The facial prominence and pharyngeal arches then undergo complex morphogenesis to form the structures of the face. AS, alisphenoid bone, F, frontal bone, FEZ, frontonasal ectodermal zone, FNP, frontonasal prominence, H, hyoid bone, I/S, incus and stapes, M, mandible, MX, maxilla, N, nasal bone, PA, pharyngeal arches, r, rhombencephalon, S, squamosal, Z, zygomatic bone.

Underpinning the complex morphogenesis of head and facial development is a population of cells called neural crest cells (NCC). Considered a vertebrate-specific cell type, NCC are transiently generated during the neurulation phase of embryogenesis which corresponds to about 3–4 weeks of human development. Specified in the neural ectoderm along nearly the entire length of the embryo, NCC undergo an epithelial-to-mesenchymal transition (EMT), which facilitates their delamination and migration throughout the primitive head. Cranial NCC give rise to the chondrocytes and osteoblasts of cartilage and bone, the fibroblasts of connective tissue, the odontoblasts in teeth, the sensory neurons and glia in the peripheral nervous system, and the pigment cells in the skin (Le Douarin and Kalcheim, 1999; Bronner and LeDouarin, 2012). Ultimately, there is barely a tissue or organ throughout the entire body that does not receive a contribution from NCC. Given this remarkable differentiation capacity, NCC have been described as the fourth primary germ layer (Hall, 1999). The specification of neural crest cell progenitors is thought to occur during gastrulation in the neural plate border (Trainor and Krumlauf, 2001, 2002; García-Castro et al., 2002; Basch et al., 2006; Prasad et al., 2020). This territory is defined as the junction between the neural ectoderm and the surface ectoderm and in chick embryos is demarcated by the expression of *Pax7* (Basch et al., 2006). During neurulation, the two halves of the neural ectoderm or neural plate elevate, converge and fuse to form a neural tube, which is the precursor of the central nervous system (Figure 1A). At the same time, neural crest cells are induced to form in the dorsolateral aspect of the neural plate in response to signals from the surrounding ectoderm, mesoderm, and endoderm. Considerable evidence has shown that signaling cascades mediated by BMP (Bone Morphogenetic Protein), FGF (fibroblast growth factor), and Wnt (Wingless/Int) play central roles in neural crest induction, although the importance and spatiotemporal regulation of these individual signaling pathways varies depending on the species (Bae and Saint-Jeannet, 2014). The potential reasons for, and significance of, these species-specific differences have been previously discussed (Barriga et al., 2015).

Irrespective of which signaling pathways are involved, the formation of NCC involves tremendous cytoskeletal changes. During EMT, adjoining neuroepithelial cells lose their intracellular tight junctions, adherens junctions, and apicobasal polarity, and acquire focal adhesions, become polarized and migratory (Taneyhill and Padmanabhan, 2014). These changes in cell adhesion are mediated in part by a “Cadherin switch” in which E-cadherin expression is downregulated in concert with N-cadherin upregulation (Hatta and Takeichi, 1986; Coles et al., 2007). A number of transcription factors including members of the Snail, Zeb and Twist protein families play critical roles in NCC EMT (Nieto et al., 1994; Van De Putte et al., 2003; Coles et al., 2007; Mayor and Theveneau, 2012) in part through directly repressing the transcriptional activity and function of E-cadherin (Cano et al., 2000). However, again there are species-specific differences in the absolute requirement and functions of these transcription factors in NCC EMT (Barriga et al., 2015). Nonetheless, the induction, EMT, delamination, migration, and differentiation of NCC depends on integrated gene regulatory networks in which many genes and signaling pathways exhibit reiterative functions.

Neural crest cells arise progressively in an anterior-posterior manner along nearly the entire neuroaxis of the embryo and are classified into cranial, cardiac, trunk, vagal, and sacral NCC axial populations. Of particular relevance in this review are the cranial NCC, which generate most of the craniofacial skeleton in vertebrates. Cranial NCC delaminate from the diencephalon (posterior forebrain), mesencephalon (midbrain), and rhombencephalon (hindbrain) and give rise to the majority of the bone, cartilage and connective tissue of the head and face (Figure 1B) (Achilleos and Trainor, 2015). The most anterior cranial NCC migrate collectively and populate the frontonasal and periocular regions, where they contribute to the nasal and frontal bones, the meninges underlying the calvarial bones and most of the suture mesenchyme separating the skull bones. The posterior cranial NCC migrate in discrete segregated streams and populate the pharyngeal arches (Osumi-Yamashita et al., 1994; Tam and Trainor, 1994; Trainor and Tam, 1995; Trainor et al., 2002), where they differentiate into the upper and lower jaw, middle ear, and skeletal structures in the neck (Figure 1B; Chai et al., 2000; Kulesa et al., 2010). Cranial NCC exhibit varying degrees of unipotency, bipotency and multipotency and are capable of differentiating into neurons and glia of the peripheral nervous system, as well as osteochondroprogenitors (Baroffio et al., 1991; Le Douarin et al., 2004; Dupin et al., 2010; Baggiolini et al., 2015). Migrating neural crest cells express *Sox10* and *Foxd3*, and the activity of these factors persist in cranial NCC destined for neuroglial differentiation, but are switched off in osteochondroprogenitors (Bhatt et al., 2013). Conversely, *Sox9*, a master regulator of chondrogenesis is expressed in cranial NCC destined for cartilage and bone differentiation but is switched off in neuroglia progenitors (Trainor and Krumlauf, 2001; Dash and Trainor, 2020).

Several mechanisms may account for the ability of NCC to differentiate into diverse cell types and tissues. If the fate of NCC was predetermined at the time of induction, NCC would comprise a heterogeneous mixture of unipotent progenitor cells, with each giving rise to a singular distinct cell type. Their differentiation would therefore be primarily dependent upon intrinsic signals (Bhatt et al., 2013). However as noted above, NCC exhibit varying degrees of cell fate potency, and therefore depend upon a combination of intrinsically expressed factors in concert with extrinsic signals emanating from the tissues they contact during their migration to undergo their proper spatiotemporal patterns of differentiation (Trainor and Krumlauf, 2001; Trainor, 2003, 2013; Trainor et al., 2003; Crane and Trainor, 2006). These key principles of NCC heterogeneity, potency, and plasticity which were determined through classic embryology, lineage tracing, and transplantation studies have been further substantiated by more recent genetic and molecular analyses such as single cell RNA-sequencing (Morrison et al., 2017; Shang et al., 2018; Soldatov et al., 2019). The remarkable lineage potential of NCC, combined with a limited capacity for self-renewal that persists even into adult life, has raised the potential for NCC to be used in regenerative medicine (Crane and Trainor, 2006; Achilleos and Trainor, 2012; Shang et al., 2018).

Synonymous with the “new head” hypothesis (Gans and Northcutt, 1983), cranial NCC carry species-specific programming information that is integral to craniofacial development, evolution, variation, and disease (Noden, 1983; Trainor and Krumlauf, 2001; Schneider and Helms, 2003; Trainor, 2003; Trainor et al., 2003; Noden and Trainor, 2005). Proper craniofacial development therefore requires that an embryo generates and maintains a sufficient number of NCC that proliferate, survive, migrate, and differentiate in the correct spatiotemporal manner. Perturbation of any one of these phases of NCC development can lead to variable craniofacial malformations. A growing body of evidence suggests that NCC are particularly sensitive to exogenous environmental stressors such as diabetes, oxidative stress, and DNA damage (Sakai and Trainor, 2016; Sakai et al., 2016; Kitami et al., 2018; Yamaguchi et al., 2021). We postulate that the interactions between these exogenous stressors and genetic risk factors for individual craniofacial malformations compromise NCC viability, thus contributing to the phenotypic variation observed in many craniofacial syndromes. To illustrate this concept, we discuss craniofacial syndromes with well recognized broad phenotypic variation that are known to be influenced by diabetes, oxidative stress, and DNA damage.

## Gene-Environment Interactions Influence Phenotype Variability in Different Craniofacial Disorders

### Treacher Collins Syndrome

Treacher Collins syndrome (TCS, OMIM number 154500) is a prime example of the considerable phenotypic variability characteristic of congenital craniofacial disorders. Extensive inter- and intra-familial variation is a striking feature of the condition (Dixon et al., 1994; Marres et al., 1995; Jones et al., 2008). TCS is characterized by anomalies of the head and face, including hypoplasia of the facial bones, especially the mandible and zygomatic complex, which may result in dental malocclusion. The palate is often high-arched or cleft (Poswillo, 1975). Other clinical features of TCS include alterations in the shape, size, and position of the external ears, which are frequently associated with atresia of the external auditory canals and anomalies of the middle ear ossicles (Edwards et al., 1996). In the most extreme cases of TCS, the constellation of craniofacial anomalies can result in a compromised airway leading to perinatal death (Edwards et al., 1996). In contrast, some individuals can be so mildly affected that it is difficult to establish an unequivocal diagnosis. It is therefore not uncommon for mildly affected TCS patients to be diagnosed retrospectively, after the birth of a more severely affected child or sibling.

TCS occurs with an estimated incidence of 1 in 50,000 live births (Carey, 1992; Twigg and Wilkie, 2015) and is caused primarily by mutations in the *TCOF1* gene. However, TCS is also associated with mutations in *POLR1B*, *POLR1C* and *POLR1D*. With respect to *TCOF1* the mode of inheritance is autosomal dominant, although very rare cases of autosomal recessive mutations have been observed (Dixon et al., 1996; Edwards et al., 1997). For *POLR1B*, all mutations to date appear to be autosomal dominant, whereas for *POLR1C* they are autosomal recessive (Dauwerse et al., 2011; Ghesh et al., 2019; Sanchez et al., 2020). In contrast, both autosomal dominant and recessive mutations in *POLR1D* have been reported in association with TCS (Dauwerse et al., 2011).

Hundreds of family-specific mutations including deletions, insertions, splice site, missense, and nonsense mutations have been identified in the *TCOF1* gene (databases.lovd.nl/shared/genes/TCOF1). However, irrespective of the position of the mutation, or the type of mutation, or whether the mutation is maternally or paternally inherited, these factors apparently have no impact on the severity of the TCS condition, and there does not appear to be any significant sex-based difference in the effect of a mutation on male vs. female offspring. Although the penetrance of genetic mutations underlying TCS is high, approximately 60% of cases arise randomly or spontaneously as a result of a *de novo* mutation in a family without a history of the disorder. The high degree of variability in which individuals with TCS are affected, together with the high rate of *de novo* mutations and the absence of a strong genotype-phenotype correlation, renders the provision of genetic counseling complicated, particularly where the diagnosis of an affected child’s parents is equivocal (Trainor et al., 2009).

*TCOF1* encodes the nucleolar phosphoprotein Treacle, which together with Upstream Binding Factor (UBF) stimulates transcription of ribosomal DNA by RNA Polymerase I (Valdez et al., 2004). POLR1B is a catalytic core subunit of RNA Polymerase I, whereas POLR1C and POLR1D comprise core subunits of RNA Polymerases I and III. Each of these factors play essential roles in rDNA transcription, which is the first step and a rate limiting step in ribosome biogenesis. Ribosome biogenesis is the process of making ribosomes, the ribonucleoprotein machines that translate mRNA into protein, thus synthesizing proteins within all cells. Since ribosomes underpin protein production, their synthesis consumes a cell’s metabolic capacity, and ribosome biogenesis is therefore tightly integrated with and regulates many cellular processes including proliferation, survival, growth, and differentiation. Interestingly, deficiencies in rDNA transcription and ribosome biogenesis result in the activation and stabilization of p53 and ultimately cell death (Rubbi and Milner, 2003). Loss-of-function mouse and zebrafish models of *TCOF1*, *POLR1B*, *POLR1C* or *POLR1D* homologs exhibit extensive p53 dependent neuroepithelium and neural crest cell apoptosis, which presages hypoplasia of the craniofacial skeleton, mimicking the characteristic features of TCS in humans (Dixon et al., 2006; Jones et al., 2008; Noack Watt et al., 2016; Sanchez et al., 2020). Furthermore, pharmacological or genetic inhibition of p53-dependent apoptosis prevents TCS in animal models (Jones et al., 2008; Noack Watt et al., 2016). TCS is therefore primarily associated with perturbation of rDNA transcription and a subsequent deficiency in the ribosome biogenesis and protein translation necessary for neuroepithelial neural crest cell proliferation and survival (Dixon et al., 2006; Noack Watt et al., 2016).

The p53 inhibition rescue of TCS occurred without restoration of ribosome biogenesis (Jones et al., 2008). This led to the suggestion that Tcof1/Treacle may also perform non-rDNA transcription and ribosome biogenesis associated functions during development. Treacle was subsequently found to directly interact with the MRNM (MDC1-RAD50-NBS1-MRE11) complex (Sakai et al., 2016), which mediates the double-stranded DNA damage response. In support of this observation, two other studies focused on the role of NBS1 in response to DNA damage induced by laser microirradiation in cultured cells, identified TCOF1/Treacle as a direct binding partner of NBS1 (Ciccia et al., 2014; Larsen et al., 2014; Sakai et al., 2016). Collectively, this implied that TCOF1 might play a key role in the response to DNA damage via the MRNM complex. Treacle was subsequently shown to localize to sites of DNA damage and *Tcof1*^+/−^ embryo-derived mouse embryonic fibroblasts (MEFs) exhibited a delay in DNA damage repair (Sakai et al., 2016). Furthermore, p-ATM was observed to be upregulated in *Tcof1*^+/−^ embryos compared to control littermates, and γ-H2AX, p-Chk2 and p53 were activated in the same neuroepithelial cells undergoing apoptosis *in vivo* in *Tcof1*^+/−^ embryos (Sakai et al., 2016). Treacle-dependent NBS1 translocation regulates silencing of RNA polymerase I-dependent rRNA transcription upon DNA damage (Ciccia et al., 2014; Larsen et al., 2014; Sakai et al., 2016), and interestingly in the absence of Treacle, BRCA1 no longer localizes to sites of DNA damage (Sakai et al., 2016). These results provided direct evidence that *TCOF1*/Treacle functions in the DNA damage response and repair pathway *in vivo* (Sakai et al., 2016). Furthermore, it connected deficient DNA damage repair and the p53 dependent apoptotic elimination of cranial NCC in *Tcof1*^+/−^ embryos as a component of the cellular and developmental mechanisms underlying the pathogenesis of TCS.

**FIGURE 2:**
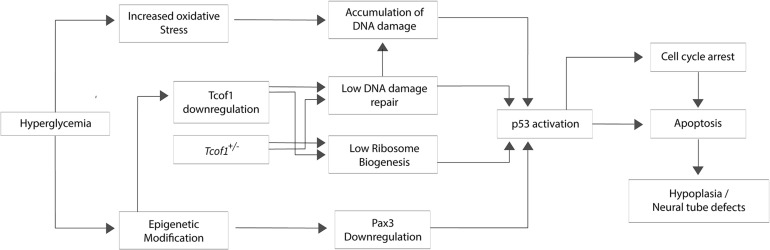
Potential mechanisms for hyperglycemia, oxidative stress, and DNA damage in the pathogenesis of Treacher Collins syndrome. The hyperglycemic environment characteristic of maternal diabetes can lead to oxidative stress and epigenetic modification. Oxidative DNA damage and aberrant *Pax3* silencing lead to p53 activation which induces apoptosis particularly within neuroepithelial cells and neural crest cells, resulting in neural tube defects or hypoplasia of neural crest-derived tissues.

Neuroepithelial cells including progenitor neural crest cells endogenously generate high levels of reactive oxygen species (ROS) compared to other tissues during embryogenesis (Sakai et al., 2016). Furthermore, exposing wild-type embryos to strong oxidants such as 3-nitropropionic acid or H_2_O_2_ induces apoptosis specifically in the neuroepithelium and progenitor neural crest cells. Thus, not only do these cells naturally exist in a highly oxidative state, they are also particularly sensitive to exogenous ROS (Sakai and Trainor, 2016; Sakai et al., 2016). Furthermore, mutations in genes critical for responding to and repairing DNA damage, would increase the sensitivity to exogenous ROS as is the case in *Tcof1*^+/−^ embryos (Sakai et al., 2016). Conversely, antioxidant supplementation provided a therapeutic avenue for ameliorating or even preventing ROS induced DNA damage phenotypes. Treating *Tcof1*^+/−^ embryos *in utero* with a strong antioxidant such as N-acetylcysteine is able to clear the ROS, thereby preventing DNA damage, p53 activation and apoptosis. Consequently, about 30% of antioxidant treated *Tcof1*^+/−^ embryos were fully rescued and morphologically indistinguishable from their wild-type littermates (Sakai et al., 2016). Thus, *Tcof1*/Treacle plays an essential role in protecting neuroepithelial and neural crest cells from endogenous and exogenous oxidative stress-induced DNA damage during normal craniofacial development. Consistent with this idea, a SILAC analysis of oxidative stress-mediated proteins in human pneumocytes revealed a potential role for Treacle in oxidant defense (Duan et al., 2010). Given that the *in utero* gestational environment generates and is subjected to dynamic levels of oxidative stress that fluctuate during an individual pregnancy and vary between pregnancies, these results imply that differential levels of oxidative stress contribute to the inter- and intra-familial variability in craniofacial anomalies characteristic of TCS (Figure 2).

The inter-and intra-familial phenotypic variability observed in association with TCS in humans can be reproduced experimentally in mice with mutations in *Tcof1* on different genetic backgrounds (Dixon and Dixon, 2004). This illustrates the potential for complex interactions between *Tcof1* and intrinsic background-specific modifier genes, or extrinsic environmental factors, in modulating phenotype variability and severity. In fact, it is tempting to speculate that a combination of endogenous background specific levels of *TCOF1*/Treacle, genetic modifiers and levels of ROS collectively determines TCS phenotypic outcomes.

### Holoprosencephaly

A complex genotype-phenotype relationship has also been observed in holoprosencephaly (HPE; OMIM number 236100), which affects approximately 1 in 16,000 live births (Geng and Oliver, 2009). HPE is a structural brain malformation characterized by incomplete or absent division of the forebrain (prosencephalon) into two cerebral hemispheres, which normally occurs by the 5th week of gestation (Golden, 1999; Kruszka and Muenke, 2018). HPE may present as an isolated phenotype (non-syndromic) or as part of a syndrome (syndromic), the most common of which include Trisomy 13 and 22, as well as Smith-Lemli-Opitz syndrome and Hartsfield syndrome (Kruszka and Muenke, 2018). Non-syndromic HPE is commonly associated with pathogenic variants in one of four principal genes including *SHH*, *ZIC2*, *SIX3*, and *TGIF* (Roessler et al., 1996, 2009; Solomon et al., 2009; Taniguchi et al., 2012). Other genetic loci, such as *GLI2*, *CDON* (also known as *CDO*), *FGF8*, and *DISP1* have also been associated with HPE or HPE-like phenotypes at lower frequency (Roessler et al., 2003, 2009; Bae et al., 2011; Hong et al., 2018).

Similar to *TCOF1* mutations in TCS, the phenotypic consequences of loss-of-functions mutations in these HPE associated loci correlate with a spectrum of facial malformations, ranging from non-lethal microforms such as hypotelorism, midfacial hypoplasia, and a single maxillary incisor, to an extremely severe form characterized by cyclopia and proboscis (Solomon et al., 2010). Depending on the degree of separation between the cerebral hemispheres, HPE is generally classified into four main subtypes: alobar, semilobar, lobar, middle interhemispheric (Solomon et al., 2010), together with a new classification called septopreoptic variant (Hahn et al., 2010). In alobar HPE, the lateral and third ventricles are completely fused, resulting in the absence of midline separation between cerebral hemispheres. Semilobar HPE occurs when the interhemispheric fissure, or the dividing line between left and right side of the brain, is only present posteriorly. In the less severe lobar HPE, the cerebral hemispheres are mostly divided except for the rostral portion of the frontal cortex. Meanwhile, the middle interhemispheric variant of HPE is characterized by the presence of interhemispheric fissure only in the anterior and posterior part of the brain, which results in medial cerebral hemispheres fusion. Lastly, the septopreoptic variant is considered the mildest form of HPE, with fusions only present in the septal and/or preoptic regions of the brain (Petryk et al., 2015). In clinical settings, many patients with HPE fall within the border zone of neighboring subtypes, and thus HPE is postulated to exist as a continuum of phenotypes rather than discrete subtypes (Hahn and Barnes, 2010).

The pathogenesis of HPE is complex and involves both genetic causes and environmental risk factors. HPE occurs due to defective development of the axial midline, which is largely orchestrated by Sonic hedgehog (SHH), BMP, FGF, WNT, Nodal, and retinoic acid signaling pathways (Grinblat and Lipinski, 2019). SHH signaling from the ventral midline is especially crucial for the outgrowth and patterning of developing brain. During embryogenesis, the brain is partitioned into prosencephalon, mesencephalon, and rhombencephalon (Figure 1B). While all three regions undergo further compartmentalization, the most relevant region in HPE pathogenesis is the prosencephalon or forebrain, which is further divided anteriorly into the telencephalon and posteriorly into the diencephalon. Unlike TCS, the craniofacial phenotypes associated with HPE do not come primarily from excessive apoptosis within the neural tube but instead are consequences of the molecular reprogramming of SHH signaling activity (Cordero et al., 2004; Richbourg et al., 2020). Nonetheless, apoptosis within cranial NCC due to aberrant Shh signaling can add to the severity of HPE (Cordero et al., 2004).

SHH plays a key role in coordinating dorsoventral polarity of the forebrain by establishing ventral identity in the neural tube during early embryogenesis (Ericson et al., 1995). Hedgehog (HH) proteins undergo lipid modifications and are anchored to the membrane of the producing cells prior to secretion. After being released from the cell membrane by Dispatched (DISP1), HH then binds to its receptor PTCH, which subsequently relieves the inhibition of SMO facilitating signaling through the GLI protein family (Burke et al., 1999; Ruiz and Altaba, 1999; Denef et al., 2000). Other HH-binding proteins, such as BOC, CDO, and GAS1 may act as co-receptors to enhance SHH signaling activity (Tenzen et al., 2006; Allen et al., 2007). Considering the central role that SHH signaling plays during midfacial development, it is perhaps unsurprising that mutations in *SHH* loci are the most common genetic cause of HPE in humans (Roessler et al., 2018). However, individuals with *SHH* mutations display incomplete penetrance, with only about 37% of carriers actually developing HPE (Roessler et al., 1996). Similarly, mutations in other *SHH*-related genes such as *GLI2* and *ZIC2* lead to HPE with variable severity. This indicates that haploinsufficiency for the respective genes alone is insufficient to elicit the full spectrum of HPE phenotypes (Petryk et al., 2015).

**FIGURE 3:**
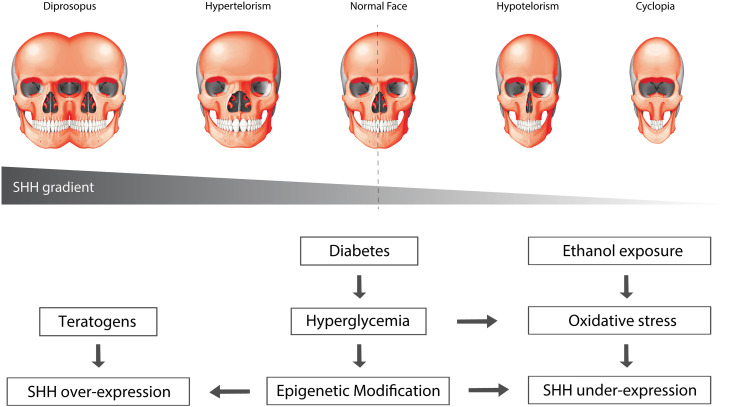
Environmental factors can affect SHH signaling. Oxidative stress and epigenetic modification can alter the levels of SHH signaling. Over-activation of SHH results in widening of the midline, leading to phenotypes such as hypertelorism and diprosopus, or facial duplication. Conversely, suppression of SHH signaling results in narrowing of the midline, leading to hypotelorism and cyclopia, phenotypes that are commonly associated with holoprosencephaly.

The variable severity of HPE may be associated with the time at which HH signaling is disrupted (Cohen, 2006), or a dose-dependent decrease in signaling activity. In 1908, anatomist Harris Wilder postulated the “Morphology of Cosmobia” where he speculated that a spectrum of symmetrical anomalies of the face was due to “*some modification in the germ itself, leading the organisms to develop in accordance with laws as definite and natural, though not as usual, as those governing normal development*” (Wilder, 1908). This spectrum of facial anomalies in effect corresponds to a gradient of Shh signaling activity, where elimination or a significant reduction in Shh signaling leads to cyclopia, a severe form of HPE characterized by a single median eye and proboscis, while in contrast, increased Shh signaling can result in facial duplication (Wilder, 1908; Figure 3). In support of this idea, work in chick embryos has shown that varying the level of Shh signaling affects the induction and spatial organization of the frontonasal ectodermal zone (FEZ) (Cordero et al., 2004), and alters dorsoventral patterning of the forebrain (Brugmann et al., 2010), each of which results in significant changes in facial appearance.

Animal models provide evidence for a functional threshold level of Shh signaling below which HPE phenotypes are always severe. In mice, homozygous mutation of *Shh* results in cyclopia and proboscis, leading to embryonic lethality, whereas *Shh* heterozygous mice are morphologically normal (Chiang et al., 1996). Genetic background also has a major effect on the penetrance of HPE phenotypes in mice. For instance, a homozygous mutation of *Cdo* on a 129S6/SvEvTac background results in mild facial microforms of HPE, whereas on a C57BL/6NTac background results in phenotypes similar to semilobar HPE (Chiang et al., 1996). Other intrinsic signaling pathways affecting the level of *Shh* expression may also contribute to HPE phenotypic variation. For example, mutations of *Tgif*, which maintains the balance between Shh and its antagonist Gli3 (Taniguchi et al., 2012), result in a more severe HPE phenotype when coupled with *Shh* haploinsufficiency compared to phenotypes from individual mutations alone (Chiang et al., 1996). Tgif protein can bind to a retinoic acid response element (RARE) in Cyp26a1, which plays a critical role in anterior-posterior patterning of the forebrain through the degradation of retinoic acid (Gongal and Waskiewicz, 2008). Sub-teratogenic doses of retinoic acid, which are often prescribed to treat skin conditions, thereby sensitize embryos to *Tgif* mutations (Bartholin et al., 2006). This supports the notion of a Shh threshold, where any additional stress, be it from genetic factors or the environment, can lower *Shh* expression below the level at which HPE always manifests (Bartholin et al., 2006).

Major environmental risk factors implicated in human HPE include maternal diabetes and ethanol exposure, which converge on SHH signaling. Around 1–2% of infants born from diabetic mothers develop HPE and women with gestational diabetes have twice the risk for HPE compared to control mothers (Petryk et al., 2015). Maternal hyperglycemia can disrupt the oxidant-antioxidant balance in the embryos and increase oxidative stress, increasing the severity of HPE (Zhao and Reece, 2005; Figure 3). Similarly, ethanol exposure impairs *Shh* expression and causes defects in midline development. Ethanol activates PKA, a negative regulator of Shh signaling, in the anterior prechordal mesendoderm during midline specification, and subsequently induces apoptosis (Lepage et al., 1995; Pan and Rubin, 1995; Hammerschmidt et al., 1996; Ahlgren and Bronner-Fraser, 1999; Aoto et al., 2008). Both ethanol-induced cranial neural crest cell death and associated craniofacial growth defects can be rescued by exogenous Shh, suggesting that craniofacial anomalies resulting from fetal alcohol exposure are caused at least partially by loss of Shh and its effects on neural crest cell survival (Ahlgren et al., 2002). In addition, dietary antioxidant supplementation can prevent the abolition of *Shh* expression as well as apoptosis in a dose-dependent manner. This indicates that oxidative stress can downregulate Shh expression and may contribute to the phenotypic variability observed in *SHH* heterozygous patients (Aoto et al., 2008). More recently, ethanol was shown to synergize directly with *Cdo* mutations to suppress *Shh* expression and elicit severe HPE on a 129S6 background, which would otherwise only exhibit a mild phenotype (Hong and Krauss, 2017). Interestingly, antioxidant treatment did not alter the frequency or severity of HPE phenotypes in these mice despite normalization of ROS levels. These conflicting results suggest that ethanol’s teratogenicity may occur via multiple mechanisms depending on the genetic background and developmental context.

With respect to the HPE continuum, a functional ceiling is likely to exist where Shh signaling above a certain level can induce replication stress and DNA damage. Consistent with this idea, overexpression of the Shh co-receptor gene *BOC* results in elevated Shh-induced replication stress and DNA damage, which increases the incidence of *Ptch* loss-of-heterozygosity, leading to constitutive activation of Shh signaling (Mille et al., 2014). It is well-known that *Ptch* gain-of-function can cause HPE due to ventralization of the neural tube and incorrect specification of the forebrain (Goodrich et al., 1999; Mullor and Guerrero, 2000), however, it has yet to be determined whether rescuing DNA damage can ameliorate the effect of Shh over-activation in this case. Aside from replication stress, mutations resulting in excessive Shh signaling lead directly to increased proliferation of neural crest cells, which can manifest as hypertelorism and frontonasal dysplasia (Mille et al., 2014). Furthermore, mouse embryos derived from dams with streptozotocin-induced diabetes exhibit expanded *Shh* expression in the ventral telencephalon, which leads to a phenotype similar to the middle interhemispheric variant of HPE (Brugmann et al., 2010). Taken together, the variable expressivity of similar HPE gene mutations can be attributed to co-morbid genetic interactors and environmental modifiers.

## Diabetes, Oxidative Stress and DNA Damage Affect Craniofacial Development and Modulate Phenotype Varibility in Craniofacial Syndromes

### Hyperglycemia in Diabetic Pregnancy Alters Cellular Metabolism and Increases Oxidative Stress

Maternal diabetes involves systemic metabolic changes which can affect virtually any organ system, but the craniofacial, central nervous system and cardiovascular structures are primarily affected (Becerra et al., 1990). These diabetic pregnancy-induced malformations, collectively termed diabetic embryopathy, are thought to arise due to defects in neurulation and neural crest cell development during the early stages of organogenesis, which corresponds to approximately the first 8 weeks of human gestation (Mills et al., 1979; Li et al., 2005; Fetita et al., 2006; Loeken, 2006). The prevalence for women with either type 1 or type 2 diabetes to be at high risk for giving birth to babies with diabetic embryopathy (Towner et al., 1995), suggests a fundamental causal role for hyperglycemia and increased glucose uptake to the embryo via glucose transporters (Loeken, 2020).

Excessive glucose metabolism increases oxidative phosphorylation (OXPHOS) and the production of reactive oxygen species (ROS), which induces a state of oxidative stress if not balanced by increased antioxidant capacity (Wentzel and Eriksson, 2011; Kim et al., 2017; Loeken, 2020). Intracellular ROS such as superoxide (${\text{O}}_{2}^{-}$) is primarily produced via the oxidation of NADPH or by the partial reduction of oxygen during aerobic respiration in mitochondria. Superoxide can be converted into hydrogen peroxide (H_2_O_2_) by superoxide dismutases, which then either oxidizes cysteine residues on proteins or becomes converted to H_2_O by cellular antioxidant proteins such as catalase, glutathione peroxidase or peroxiredoxins. If high levels of H_2_O_2_ levels go unchecked, hydroxyl radicals (OH^–^) will form and this can result in molecular, cellular, and tissue damage during embryogenesis (Jones and Sies, 2015). However, increased oxidant status is complex, involving a combination of increased superoxide production as well as impaired free radical scavenging, although the pathways responsible for increased oxidant status have not been completely elucidated. Interestingly, early embryonic development is especially vulnerable to oxidative stress due to the lack of free radical scavenging enzymes activity (El-hage and Singh, 1990). In fact, premigratory and migratory NCC appear to be particularly at risk of free radical damage since they are deficient in superoxide dismutase and catalase activity, which are necessary for the normal inactivation of superoxide, hydrogen peroxide and hydroxyl radicals (Davis et al., 1990; Chen and Sulik, 1996). This is consistent with the neuroepithelium from which NCC originate, existing in a highly oxidative state and being particularly sensitive to exogenous oxidative stress (Sakai et al., 2016), thus indicating that cranial NCC possess lower tolerance to the detrimental effect of increased ROS.

High glucose metabolism in NCC may be attributable to their rapid proliferation and motile nature, reminiscent of the Warburg effect in cancer metastasis (Warburg, 1956). Actively dividing cells favor glucose metabolism through aerobic glycolysis to produce biomass. In contrast, terminally differentiated cells rely on OXPHOS to generate energy more efficiently from glucose (Warburg, 1956). Cellular glucose metabolism thus alternates between aerobic glycolysis and OXPHOS depending on the stage of development. During EMT, neural crest cells undergo similar cytoskeletal and molecular changes observed in metastatic tumor cells where aerobic glycolysis is increased to serve the anabolic demand of proliferation. Enhanced aerobic glycolysis promotes the Yap/Tead pathway that is necessary for cell delamination during EMT (Bhattacharya et al., 2020). Conversely, the decay of glycolytic activity and increased OXPHOS correlate with the loss of mesenchymal motility (Warburg, 1956), suggesting that hyperglycemia may accelerate the differentiation of neural crest-derived tissues through preferential switching to OXPHOS. Additionally, hyperglycemia-induced oxidative stress leads to the oxidation of cholesterol, lipids, and proteins, which have been proposed to contribute to the pathology of Smith-Lemli-Opitz syndrome (Richards et al., 2006) and thus may add to the phenotypic variability of HPE. Since proper Shh gradient formation is dependent upon cholesterol modification, oxidation of cholesterol can directly impact Shh signaling and impair neural tube patterning (Guerrero and Chiang, 2007; Porter and Herman, 2011). More studies are still needed to understand whether untimely switching to OXPHOS and increased cholesterol oxidation contribute to increased risk of craniofacial malformation or variation in craniofacial development. However, it is clear that improper fluctuations of glucose metabolism in diabetic embryopathy can adversely affect NCC EMT and migration as well as neural tube patterning, resulting in craniofacial malformations.

### Hyperglycemia-Induced Oxidative Stress Leads to Epigenetic Modifications and Altered Gene Expression

One of the negative effects of excess ROS is that it can disrupt key signaling events during cellular differentiation, resulting in structural abnormalities (Kemp et al., 2008). In fact, many developmental genes exhibit specific sensitivities to hyperglycemic conditions and changes in the cellular redox state (Fetita et al., 2006; Wu et al., 2012). This may be due in part to the presence of binding sites for transcription factors involved in response to oxidative stress in their promoters (Pavlinkova et al., 2009). These genes which were identified under the conditions of maternal diabetes, and in the absence of genetic alterations, are therefore subject to gene-environment interactions in their response to the intrauterine environment of a diabetic pregnancy. Further evidence indicates that environmental factors can perturb gene regulation, which may affect gene dosage variability in individuals from different genetic backgrounds (Phelan et al., 1997). For instance, both diabetes and oxidative stress can impair Shh signaling by increasing or reducing Shh expression, which leads to defects in neural tube patterning (Pavlinkova et al., 2009). Furthermore, maternal diabetes increases the overall variability of gene expression levels in embryos, including deregulation of genes involved in Wnt, Hedgehog, and Notch signaling (Salbaum and Kappen, 2010). Additionally, diabetes-induced oxidative stress results in reduced expression of *Pax3*, which plays a major role in neuroepithelial development (Pavlinkova et al., 2009; Salbaum and Kappen, 2010). *Pax3* loss-of-function results in aberrant p53 activation, neuroepithelium and neural crest cell apoptosis, and consequently neural tube defects (Liao et al., 2004; Aoto et al., 2008) as well as malformation of structures derived from neural crest cells (Loeken, 2006; Wu et al., 2012).

Epigenetic factors, such as DNA methylation and histone modification, may also contribute to this variability through gene silencing or aberrant activation. In fact, hyperglycemia and oxidative stress were shown to trigger chromatin modifications via histone and DNA methylation. Mouse neural stem cells derived from the embryos of diabetic mothers exhibit increased global histone H3K9 trimethylation and DNA methylation, as well as decreased histone H3K9 acetylation which leads to altered miRNA expression (Shyamasundar et al., 2013; Ramya et al., 2017). Alteration of miRNA activity can impair autophagy and lead to neural tube defects such as exencephaly (Xu et al., 2013; Wang et al., 2017). The same phenomena were also observed in human neural progenitor cells in which high glucose modifies the DNA methylation pattern of neurodevelopment-associated genes, hence affecting their activity (Kandilya et al., 2020). These findings suggest that hyperglycemia can interact with genetic loci by influencing the activities of histone-modifying and DNA methyltransferase enzymes. Indeed, increased activity of DNA methyltransferase 3b (*Dnmt3b*) in mouse embryos and embryonic stem cells (mESC) of diabetic mothers result in decreased methylation of *Pax3* CpG island, which leads to silencing of *Pax3* (Wei and Loeken, 2014). More importantly, *Tcof1* and *Cdo* were shown to be deregulated in hyperglycemic embryos (Salbaum and Kappen, 2010), indicating that maternal diabetes may exacerbate TCS and HPE phenotypes by directly lowering *Tcof1* and *Cdo* expression even further. It has yet to be determined what epigenetic modification occurs within *Tcof1* and *Cdo* CpG islands, however, hyperglycemia-induced epigenetic modifications potentially underlie gene expression variability in *Tcof1*^+/−^ or *Cdo*^–/–^ mutant mice on different genetic background, which may correlate with phenotypic variability in TCS and HPE.

### A Potential Role for DNA Damage in Craniofacial Development

The rapid and sustained proliferation of premigratory and migratory NCC results in naturally high levels of ROS, which if left unchecked can lead to genotoxic stress in the form of DNA damage (Sakai and Trainor, 2016; Sakai et al., 2016). Newborns from mothers with diabetes exhibit elevated levels of 8-OHdG, which is a widely used marker for oxidative nucleotide damage (Gelaleti et al., 2015; Castilla-Peon et al., 2019), and suggests that hyperglycemia can induce DNA damage. In support of this idea, analysis of neurulation-stage mouse embryos showed that hyperglycemia increases the DNA damage marker p-H2AX, which can be suppressed by overexpression of antioxidant SOD1 both *in vitro* and *in vivo*. This indicates that the hyperglycemic environment triggers DNA damage and the DNA damage response (DDR) pathway through oxidative stress (Dong et al., 2015).

NCC-derived tissues seem to be particularly sensitive to DNA damage accumulation due to the lower antioxidant capacity and higher level of ROS in the neuroepithelium and progenitor NCC. Global treatment of mouse embryos with the mitochondrial inhibitor 3-nitropropionic acid induces ROS over-production, resulting in elevated levels of DNA damage specifically within the neuroepithelium (Sakai and Trainor, 2016; Sakai et al., 2016). Although ubiquitously expressed and central to cell survival, the localized endogenous spatiotemporal generation of ROS could render the effects of mutations in DDR genes more significant in NCC-derived tissues compared to other tissues. This is evident from the phenotypes of mutations in *BRCA1*, *MRE11*, *RAD50* and *NBS1* in humans and in mouse models. Mutations affecting the *MRE11-RAD50-NBS1* (*MRN*) protein complex are known to cause craniofacial anomalies (Chrzanowska et al., 2001; Fernet et al., 2005; Waltes et al., 2009). The MRN complex functions as a DNA damage sensor by recognizing and binding to the broken ends of DNA (Assenmacher and Hopfner, 2004; Paull and Lee, 2005; Stracker and Petrini, 2011) and thus regulates initial and sustained responses to DNA damage. Hypomorphic mutations in *NBS1* are associated with Nijmegen breakage syndrome (NBS), which is characterized by distinct facial features including a small lower jaw (Chrzanowska et al., 2012). Similarly, mutations in *MRE11* have also been shown to underlie craniofacial anomalies such as a small lower jaw, together with microcephaly as part of the rare Ataxia Telangiectasia-like disorder (Matsumoto et al., 2011). Developmentally, these phenotypes are thought to arise in part through extensive neuroepithelial apoptosis (Kobayashi et al., 2004; McKinnon, 2012), and consistent with these observations in humans, neural stem cell-specific conditional deletion of *Nbs1* and *Mre11* in mouse embryos results in microcephaly (Frappart et al., 2005).

Further support for the importance of DNA damage repair in neural crest cell and craniofacial development can be found in *BRCA1*, a tumor suppressor and a key player in the DNA damage response through its central role in homologous recombination (Frappart et al., 2005). *BRCA1* dysregulation is associated with non-syndromic cleft lip and palate, which is one of the most common human craniofacial defects (Kobayashi et al., 2013). Knockout of *Brca1* in mouse embryos results in extensive neuroepithelial cell apoptosis during the early stages of craniofacial development (Gowen et al., 1996; Hakem et al., 1996; Liu et al., 1996). Conditional deletion of *Brca1* in NCC in mouse embryos manifests in hypoplastic jaws, cleft palate, and microcephaly. NCC-derived osteogenic progenitors exhibited increased levels of γ-H2AX and p53 activation, which subsequently led to their apoptosis, resulting in cranioskeletal hypoplasia. Interestingly, the loss of *Brca1* did not affect osteogenic differentiation, indicating that Brca1-mediated DNA damage repair is critically required for osteoprogenitor survival during craniofacial development (Kitami et al., 2018; Yamaguchi et al., 2021).

**FIGURE 4:**
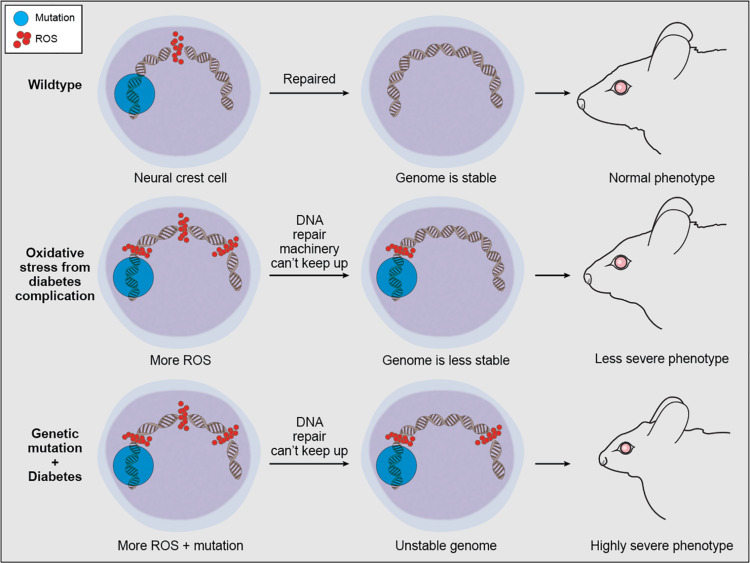
Proposed mechanism of oxidative stress contribution to phenotypic variability in craniofacial anomalies. ROS is a natural byproduct of cellular metabolism which can be scavenged by antioxidant enzymes, and ROS-induced DNA damage within normal levels can be repaired by the DDR machinery. However, continuous exogenous or environmental oxidative stress can overwhelm antioxidant enzymes and DDR capacity, leaving some ROS-induced DNA damage unrepaired. This unrepaired DNA damage can compound the detrimental effects of genetic mutations associated with craniofacial malformations.

These findings illustrate the importance of maintaining genome integrity during NCC development and help to account for why disruptions in a central process such as the DNA damage response can result in tissue-specific developmental defects. Given that the neuroepithelium exists naturally in a highly oxidative state, which lowers its threshold for oxidative stress-induced p53 activation compared to other tissues (Sakai et al., 2016), suppressing p53 function should in theory offer an avenue for the prevention of some craniofacial malformations. Indeed, both pharmacological and genetic inhibition of p53 function can decrease neuroepithelial apoptosis and rescue animal models of TCS (Jones et al., 2008), open neural tube defects (Pani et al., 2002), and HPE (Billington et al., 2011). Preventing p53 activation through maintenance of proper physiological levels of ROS can therefore help avoid the detrimental effects of DNA damage. In support of this idea, NAC antioxidant supplementation ameliorated the TCS phenotype in *Tcof1*^+/−^ mouse embryos via the diminishment of γ-H2AX, p-Chk2, and p53 (Sakai et al., 2016). Similarly, several studies have shown that administration of antioxidants, particularly vitamins C or E, or overexpression of superoxide dismutase reduce the incidence of developmental defects in experimental models of intrauterine diabetes and hyperglycemia (Aoto et al., 2008). Taken together, these data reveal the importance of redox homeostasis for proper developmental signaling and cell viability. Redox homeostasis is maintained through a fine balance between oxidants and antioxidants and when an imbalance occurs prolonged oxidative stress can induce genotoxic stress in the form of DNA strand breaks. Maternal diabetes, smoking and alcohol consumption during pregnancy are all factors known to increase maternal ROS levels, which can be damaging to the genomic DNA of embryos (Ornoy, 2007). Thus in the absence of key pathways for detoxifying ROS or DNA damage repair, persistent hyperglycemia-induced oxidative stress can have embryopathic consequences (Wells et al., 2010) or exacerbate the phenotypic severity caused by a particular genetic mutation (Figure 4). Although the full extent of oxidative stress-induced DNA damage remains to be elucidated, multiple studies have indicated that insufficient DNA damage repair capacity, particularly within premigratory and migratory neural crest cells, can lead to craniofacial malformations (Ornoy, 2007). More importantly, this suggests that oxidative stress-induced DNA damage can underpin gene-environment interactions and influence the variable phenotypic severity observed in many craniofacial disorders and syndromes.

## Concluding Remarks and Future Perspectives

The anatomical complexity of the craniofacial complex coupled with the initiation of its development during early embryogenesis renders the head and face prone to malformation. One of the biggest clinical challenges in craniofacial biology is the frequent lack of accurate genotype-phenotype correlation. This illustrates the need for more detailed quantitative phenotyping to accurately capture the full spectrum of variation for an individual craniofacial syndrome, but it also implies that both genetic and environmental factors contribute to the etiology and pathogeneses of craniofacial anomalies. One of the biggest risk factors for increased severity in craniofacial disorders is maternal diabetes (Ewart-Toland et al., 2000; Chappell et al., 2009). Hyperglycemia, which is the hallmark of diabetes, disrupts cellular metabolism, induces over-production of reactive oxygen species (ROS), and dysregulates genes involved in craniofacial development. We postulate that the detrimental effect of any candidate mutation causing a craniofacial anomaly will be amplified by oxidative stress-induced DNA damage in the neuroepithelium and NCC (Figure 4). TCS is a prime example of this synergistic interaction. Haploinsufficiency of *Tcof1* not only disrupts rDNA transcription and ribosome biogenesis, which activates p53 thereby diminishing NCC proliferation and survival, but haploinsufficiency of *Tcof1* also perturbs the DNA damage response and affects the ability of *Tcof1*^+/−^ embryos to survive under endogenously high levels of oxidation (Dixon et al., 2006; Jones et al., 2008; Sakai et al., 2016). This demonstrates that DNA damage-inducing stress in the gestational environment, such as in the case of maternal diabetes and alcohol exposure, or modifier mutations in DNA damage response and repair genes could therefore affect phenotypic variability and compound TCS severity.

Although the complete mechanisms underpinning the teratogenic effects of maternal diabetes during pregnancy on development are not yet fully understood, it is clear that diabetes-induced oxidative stress, and oxidative stress-induced DNA damage, impacts neuroepithelial and neural crest cell survival and patterning, resulting in significant craniofacial dysmorphogenesis (Aoto et al., 2008). Optimizing maternal metabolic control in the first trimester of gestation during which neurulation and neural crest cell formation and migration occur is therefore critical for protecting newborns against oxidative damage and to ensure normal craniofacial morphogenesis. Suppression of p53-dependent apoptosis appears to be key in preventing many craniofacial anomalies by ensuring survival of neural crest cells throughout development. Although promising, inhibition of p53 poses an unacceptably high risk due to its role as a tumor suppressor. Thus, circumventing p53 activation by maintaining the correct physiological levels of oxidation is a potential avenue for preventing or reducing the severity of craniofacial anomalies. It is important to note however, that lowering ROS too far can pose a cytostatic risk where neural crest cells may not fully grow or differentiate, as well as increase the risk for immunosuppression within the embryo. It is also important to keep in mind that the nature of gene interactions with oxidative stress may differ according to their temporal, spatial, and biochemical context. To date, antioxidant supplementation has only been performed successfully in animal models of craniofacial disorders. Further investigation is needed to elucidate the appropriate dosage, time of administration, and side effects of antioxidant treatment as a viable means for preventing craniofacial anomalies in a clinical setting. Nonetheless, new studies should more extensively investigate the diagnostic and therapeutic value of various oxidative stress biomarkers and antioxidants to reduce oxidative tissue injury to developing newborns. Since phenotypes are frequently affected by gene-environment interactions, examining Quantitative Trait Loci using genetically diverse backgrounds under different environmental conditions may be beneficial for identifying such interactions. Using genome wide association studies (GWAS) to identify gene-environment interaction can also be advantageous for identifying high-risk subjects and improving the diagnosis of complex craniofacial diseases.

## Author Contributions

SF and PT co-drafted, edited, and revised this review article. Both authors contributed to the article and approved the submitted version.

## Conflict of Interest

The authors declare that the research was conducted in the absence of any commercial or financial relationships that could be construed as a potential conflict of interest.
